# Sustaining transformations in psychoanalysis: an interdisciplinary case study on trauma, dreams, and memory reconsolidation

**DOI:** 10.3389/fpsyg.2026.1778793

**Published:** 2026-04-28

**Authors:** Gilles Ambresin, Tamara Fischmann, Marianne Leuzinger-Bohleber

**Affiliations:** 1 Centre Hospitalier Universitaire Vaudois, Lausanne, Switzerland; 2 International Psychoanalytic University Berlin, Berlin, Germany; 3 Universitatsmedizin der Johannes Gutenberg-Universitat Mainz, Mainz, Germany

**Keywords:** chronic depression, dream analysis, early trauma, embodied trauma memory, psychoanalytic treatment, therapeutic sustained change processes

## Abstract

Chronic depression associated with early trauma remains one of the most treatment-resistant conditions in mental healthcare. Clinical and empirical findings have increasingly suggested that patients with histories of early trauma may benefit differentially from intensive psychoanalytic treatments compared to symptom-focused or short-term psychotherapies. Our conceptual article aims to elucidate the mechanisms underlying sustained therapeutic change in psychoanalysis with chronically depressed, early traumatized patients by integrating clinical psychoanalytic theory, contemporary dream research, and interdisciplinary models of memory reconsolidation. Taking a longitudinal case from the LAC Depression Study as an illustration, this study examines a patient who underwent high-frequency psychoanalysis and demonstrated enduring structural change, which remained accessible for more than a decade after treatment termination. Clinical material from the original analysis and from a later crisis intervention illustrates how embodied memories of early traumatic experiences can be discovered and understood within the transference relationship and gradually transformed through repeated analytic working-through. Particular emphasis is placed on changes in dream processes as indicators of deep psychic reorganization. Using psychoanalytically informed dream research, including the Zurich Dream Process Coding System (ZDPCS), the analysis demonstrates a shift from early trauma-dominated nightmares characterized by helplessness and an absence of a helping object to a later dream reflecting increased affect regulation, symbolic capacity, involvement, and self-agency. These transformations are conceptualized as markers of structural change rather than mere symptom reduction. This article further argues that psychoanalytic processes resonate with contemporary neurobiological models of memory reconsolidation. When embodied memories of trauma are revived within a safe and emotionally attuned analytic relationship, they may enter a labile state that allows for enduring modification and reintegration. From this interdisciplinary perspective, psychoanalysis is uniquely positioned to foster sustained change in patients with chronic depression and early trauma by enabling the re-transcription and reconsolidation of traumatic embodied memories within the analytic relationship. This conceptual integration contributes to a more profound understanding of why psychoanalysis can produce lasting therapeutic effects in a patient population traditionally considered difficult to treat.

## 1 Introduction

Chronic depression associated with early traumatic experiences poses a persistent challenge to mental healthcare. Individuals with histories of childhood neglect, emotional abuse, or other forms of severe early trauma often develop enduring disturbances in basic trust^1^ in essential object relations, one’s own self-agency, affect regulation, and social and professional skills that are not easily altered through short-term or symptom-focused interventions. Early trauma leaves a lasting influence on memory functions, unconscious fantasies, and beliefs about extreme destructiveness in object relations, reinforcing depressive vulnerability across the lifespan. For this subgroup of patients in particular, understanding which therapeutic approaches foster deep and sustainable change is of pressing clinical and scientific importance.

The question of how sustainable changes can be achieved has preoccupied psychoanalysis since its inception. We would like to contribute to the discussion of this relevant topic by presenting an interesting empirical finding from our research group. A differentiated reanalysis of data from the LAC Depression Study, which compared the results of psychoanalysis and long-term cognitive-behavioral therapy in chronically depressed patients, demonstrated that childhood trauma significantly moderates treatment response in chronic depression, with patients who reported early trauma showing greater improvement in psychoanalysis than in long-term cognitive–behavioral therapy (Krakau et al., 2024). This differential effectiveness underscores the relevance of intensive psychoanalytic approaches that directly engage the unconscious meanings of embodied memories, central unconscious fantasies, beliefs, and affects connected to important object-relations – psychological domains commonly affected by early trauma.

In this article, we discuss our hypothesis that there is a differential indication for psychoanalysis for patients with chronic depression and early trauma, because psychoanalysis offers the advantage that early, unconsciously acquired pathological (embodied) object-relationship experiences that influence thoughts, feelings, and actions in current important relationships can be studied and worked through directly in the transference relationship. Our psychoanalytically-based thesis is supported by the findings of contemporary memory research, as summarized by Lane et al. (2015). In a section concerning the implications for indications to different forms of psychotherapy, the authors write:

“Time and cost considerations aside, the technique of meeting three, four or five times per week for several years creates a special opportunity to activate old memories and observe their influence on present-day construal and emotional experiences with an emotional intensity and vividness that is difficult or impossible with other methods (Freud, 1914/1958). As such, this approach has the potential to offer something not available with other modalities that can have pervasive effects on a person’s functioning in a wide variety of social, occupational, and avocational settings” (Lane et al., 2015, p. 16) [SIC].

Lane et al. (2015) justified this thesis with their concept of memory reconsolidation. In this study, we addressed their considerations to contribute to a deeper understanding of why psychoanalysis—in contrast to cognitive behavorial therapy (CBT) and all forms of short-term therapy—is more likely to lead to sustained changes in chronically depressed, early traumatized patients. Our research group carefully analyzed detailed records of a crisis intervention 14 years after the completion of a 5-year psychoanalysis and analyzed the changes in the patient’s inner object world using two dreams as examples with the help of the Zurich Dream Process Coding System (ZDPCS). In this context, only a summary of these analyses can be presented (Section 2) (more details can be found in Ambresin et al., submitted). We discuss how the interdisciplinary perspective of recent memory research, i.e., knowledge about memory reconsolidation (Section 3), can add an additional dimension of understanding to the clinical-psychoanalytic understanding of the sustainability of transformation processes. Similarly, we gain insights by supplementing the clinical-psychoanalytic understanding of transformations in dreams in psychoanalysis with a non-clinical analysis of the patient’s dreams (Section 3.1). We refer to the dream generation model developed by Ulrich Moser and his colleagues, along with the ZDPCS based on it. In the discussion, all three perspectives are brought together in order to gain a deeper understanding of the sustainability of changes in the inner object world of chronically depressed, early-traumatized patients (Section 4).

## 2 Sustaining transformations by psychoanalysis? A clinical example of a chronically depressed, early traumatized patient

Mr. P. is one of the first patients included in the LAC depression study. As we have discussed in several articles, he belonged to the 84% of the 252 chronically depressed patients who had gone through severe early trauma (Leuzinger-Bohleber, 2024, 2025). Below is a short summary of his biography^2^.

The patient is an only child. One of the known details about his early history is that he was a “crybaby.” Over the course of the first three months of psychoanalysis, the patient characterized his parents as being loving, showing him considerable care and attention. However, over time, it became evident that both parents had severe disturbances in their empathy for their baby, likely due to their own trauma during World War II and a history of severe transgenerational trauma in their family. When he was four years old, Mr. P. was admitted to a children’s foster home, evidently founded on authoritarian, inhumane educational principles reminiscent of the National Socialist ethos. During psychoanalysis, it became absolutely clear just how traumatic his time in the home was. He lost his basic trust in his inner objects and his self-agency, and subsequently, he lived in a state of dissociation for years. In many of his dreams, he felt that he was in mortal danger after having been left alone and was full of panic-ridden anxieties and desperation (see below).

Despite his dissociative states and social isolation, Mr. P. was a good pupil in school and university. However, separations from his girlfriends triggered serious depressive breakdowns and panic attacks. He had undergone several inpatient and short-term outpatient treatments, “who gave me some relief but never helped me to really overcome my severe depression” [SIC] (Mr. P.).

This summary of his life story clearly shows the close connection between depression and trauma. As a result, Mr. P. suffered from terrible nightmares for years. At the beginning of psychoanalysis, he recounted many of them, including the following dream:

“I see a badly injured man lying by the side of the road – his intestines are hanging out and everything is drenched of blood. A helicopter appears. It is still unclear as to whether the man is being shot at, or whether he is being helped. Somebody appears and claims that the man has died. I notice that the man is still alive and that he opens his eyes and says, “Why is nobody coming to my aid?” A woman hands him the lid of a cooking pot – which he is meant to place over the wound. I wake up in a state of panic” [SIC].

In the manifest dream^3^, embodied memories of a traumatic situation are represented: the dream self is in a life-threatening, absolutely helpless situation and overwhelmed by the fear of death and panic.^4^ It can do nothing against the existential threat and danger. Nor is there any empathetic, helpful object available: the sense of basic trust in an autonomous self and a “good enough” object has collapsed.

As mentioned in the introduction, Mr. P. belongs to the group of chronically depressed patients with severe early childhood trauma who benefit more from psychoanalysis than from CBT (Krakau et al., 2024):

Over the course of 5 years of analysis, he was able to face previously dissociated traumatic pain, embodied memories, and conflicts, enabling him to emerge from pathological retreat (Steiner, 2003), reduce his depressive symptoms, live with his vulnerability, and partially regain the capacity “to work, to love and to enjoy life” (Freud, 1937/1964). A key indicator of this sustained inner transformation was the disappearance of his terrifying nightmares, which he regarded as one of the most important results of his treatment.

Fourteen years after completing psychoanalysis, Mr. P. contacted his analyst and asked her for some crisis intervention sessions. The reason for this was that his only child had moved out to pursue a demanding education abroad. This separation reawakened intense embodied memories of Mr. P.’s early separation trauma. Although he was still able to reflect on these embodied memories of early trauma, he could not prevent the fact that the acute life crisis had led him back into depressive states and psychosomatic heart problems. He was aware that the separation from his child was compounded by grief over the death of his mother, who had died shortly after her husband a year earlier, after a long illness. Another source of grief was the prospect of his imminent retirement from professional life. The finiteness of his life was drawing nearer. He felt as though he was once again in a situation where it felt “too much” for him to cope with the embodied memories of his early trauma. Therefore, he contacted his analyst to strengthen his capacity to cope with the intersection of current experiences of loss and traumatic embodied memories.

Our research team analyzed the records of the five crisis intervention sessions in detail. Here is a brief summary:

During the second intervention session, his associations to two films about severe illness, the death of a child, and a father’s transformative mourning while hiking the Camino de Santiago (a dream of his lost son) revealed his inner conflict between suicidal ideations, on the one hand, and actively “keep walking,” on the other.

“And I also had wild dreams—chaos—I still remember one dream:” I was with the builder who keeps wanting me to work for him.^5^ He wanted to sell me an old Louis Quinze furniture. I knew this was stupid. I already have lots of old stuff. Nevertheless, I bought the piece in the dream. Then I tried to tell someone about it, but a female colleague kept interrupting me (it’s mean of me to say this, but this colleague sits with me in my office at work. She does not have any children and takes great care of her parents. She always talks my ear off about her caregiving concerns. I’m under a lot of pressure at work and need to concentrate. Still, I do not want to be unsympathetic, and I want to listen to her, but sometimes I have to say, “What did you just say? I wasn’t listening!”). This comes up in the dream. I told someone about the old piece of furniture, but my colleague kept interrupting. Then another man showed up. I know him. He′s pretty pushy. He wanted to sell wine to my colleague for the funeral so they could toast. She asks me if I know the wine. I’m annoyed and reply, “What’s this all about? Who do you want to toast? Because you are glad the dead one is no longer here?” Then I wake up and think, “What a strange dream” [SIC].

His associations led to his fear of intrusive objects (the salesman, his colleague), his own aggressive impulses, his death wishes toward his daughter and parents, and his attempts to distance himself from all of this through humor. These underlying unconscious fantasies and conflicts could be elaborated on and understood in an analytical session. It also became apparent that the dream reflected the transference: in the first crisis intervention session, Mr. P. felt “interrupted” by a specific interpretation made by the analyst, just as he had been by his colleague in the dream. However, in contrast to the early phases of psychoanalysis, he was now able to actively defend himself in the manifest dream and in reality, explicitly expressing his criticism of the analyst to her.

### 2.1 Some clinical psychoanalytic remarks

Within the limited scope of this article, we must confine ourselves to only a few clinical-psychoanalytic remarks regarding possible explanations for Mr. P.’s lasting change thanks to his psychoanalysis.

At the end of his analysis, Mr. P. emphasized that no longer being tormented by nightly nightmares, being able to work and enjoy it, fulfilling his paternal duties and responsibilities toward his daughter, and understanding his bodily symptoms as rooted in childhood experiences were among the most important outcomes of his treatment.

From a psychoanalytic perspective, a further crucial outcome was his decision—during a later life crisis—to turn to his former analyst again. Severely traumatized patients often carry an unconscious conviction that no one cares about their desperate, helpless suffering; this generates profound loneliness, a central theme in Mr. P.’s analysis. Seeking help 14 years later indicates that he had built a stable alternative inner object world that counterbalanced his traumatically “destroyed” inner object world. He could now draw on embodied memories of a reliable, non-traumatogenic analytic object, making it possible for him to ask for external help.

This internal situation was mirrored in his later dream: he was no longer alone in overwhelming panic but could actively engage with themes of loss and death and defend himself, even with some humor, against intrusive figures. For traumatized patients, such an internalized helping object is existential, as trauma has shattered basic trust and self-agency. Psychoanalytic trauma research has suggested that trauma cannot be erased but can be understood and symbolized through an inner dialogue with a reliable, empathic object; Mr. P. had maintained such a dialogue for years, allowing him to work, parent, sustain friendships, and manage psychosomatic symptoms instead of being ruled by them. The separation from his adolescent daughter temporarily overwhelmed these resources, requiring a “refresh” of the inner dialogue via renewed contact with the real analyst.

Conceptually, internalizing an empathic and responsive object restores self-agency—the “fuel” of thinking, action, and self-reflection (Bohleber, 2024). Agency enables the “dual self” stance: observing one’s own traumatized history and engaging in self-reflection and insight. Mr. P. used the crisis sessions to revitalize this agency and reconnect with embodied memories of analytic insight.

We understood his enduring transformation as the result of a restructured inner object world created through long-term work on embodied traumatic memories in the transference. Internalizing a helpful, understanding, and active (non-powerless) object, along with a capable self, allowed him to continue an inner self-analytic dialogue with his traumatized self and its unconscious beliefs about destructive objects. These structural changes in object relations and self-reflective capacity both reduced depressive symptoms and altered his psychological functioning more broadly (structural change; Leuzinger-Bohleber et al., 2019). As discussed below, one way to study such a structural transformation is by examining changes in dreams over the course of psychoanalysis.

### 2.2 Understanding based on psychoanalytical dream research (referring to Moser’s model and the ZDPCS)—based on the two dreams

The dream-generation model developed by Moser and von Zeppelin (1996) integrated psychoanalytic theory with findings from memory research, Cognitive Science, and developmental research to explain dream formation and dream change. We summarized the ZDPCS and its relation to memory in a recent article (Fischmann and Leuzinger-Bohleber, 2025). Therefore, we will provide only a short summary here.

Dreaming can be understood as an internally driven mode of processing emotional memories, allowing affective experiences – especially those that are overwhelming or dissociated in waking life – to be integrated into long-term memory. According to Moser and Hortig (2019), along with contemporary psychoanalytic dream models, current stressors activate *dream complexes*, which are affect-laden, trauma-related memory structures rooted in early relational experiences. These complexes contain unbound affects and early introjects that repeatedly seek resolution.

During sleep, a *dream organizer* constructs scenarios that permit these affects to be reactivated and worked through in a tolerable form. When the emotional load becomes excessive, the dream may interrupt or shift scenes – an indicator of the dreamer’s difficulty in integrating the underlying conflict. Over time, fewer interruptions and more coherent dream narratives reflect growing symbolic capacity and improved integration of traumatic material.

From this perspective, changes in a patient’s dreams can serve as markers of deeper structural transformation: early nightmares often depict overwhelming, life-threatening states with no available helping object, while later dreams may show increased agency, relational meaning, and the capacity to symbolize loss or conflict. These transformations provide insight into how psychoanalytic treatment reorganizes embodied memories and supports enduring therapeutic change.

#### 2.2.1 ZDPCS-based interpretation of the first dream

According to the ZDPCS, the first dream (s. p. 4) is a trauma-related nightmare dominated by the *security principle*: the dream organizer focuses on reducing overwhelming negative affect rather than engaging with the underlying conflict. The associated dream complex (DCPLX) is clearly traumatic: The severely injured man represents the patient’s early separation trauma and lack of a *Helping Other*; the patient remains in a distant *observer* position, showing only rudimentary *unconscious empathy* and very low reflexivity. Because the emotional load cannot yet be integrated, the dreamwork is restricted to protective distancing and anticipations of danger, helplessness, and dependence, illustrating a fragile regulatory balance. Although the patient hopes for rescue, the dream is governed by the conviction that he cannot solve the underlying problem on his own – a direct expression of his *lack of self-agency*, which is rooted in early trauma.

#### 2.2.2 ZDPCS-based interpretation of the last dream (from the crisis intervention)

In the second dream (s. p. 6), reported 14 years after analysis, the patient no longer faces catastrophic injury or life-threatening helplessness; instead, affect is shared, manageable, and symbolically embedded in ordinary interactions. From a ZDPCS perspective, the dream is no longer governed by the security principle but rather by improved affect regulation via affect induction and equalization across several “object processors.” This allows for reciprocal involvement, mutual influence, and joint regulation, as opposed to dissociated observation. The dreamer now experiences himself as an active participant in a relational microworld, capable of receiving and metabolizing the message to “change something,” indicating increased reflexivity, emotional engagement, and the structural capacity to use internalized relational resources to manage revived traumatic tensions.

Overall, the dream reflects an increase in the dreamer’s ability for emotional involvement, mutual regulation, and symbolic processing. Affects are no longer overwhelming or disorganizing but become part of an interpersonal negotiation. This stands in contrast to the earlier dream and provides compelling evidence of sustained structural change – supporting the broader clinical picture of a patient who, years after analytic treatment, can draw on internalized relational resources to navigate revived traumatic tensions during a new crisis.

#### 2.2.3 Comparative analysis of the two dreams: evidence of sustained structural change

The contrast between the patient’s first dream at the beginning of analysis and the second dream reported fourteen years after termination demonstrates a noticeable evolution in affect regulation, symbolic capacity, and self-agency. Both psychoanalytic and ZDPCS frameworks reveal a transition from trauma-driven disorganization to flexible, dyadic regulation and meaningful involvement with others.

##### 2.2.3.1 From catastrophic helplessness to manageable engagement

Dream 1 is organized around catastrophic injury, annihilation anxiety, and a complete absence of an effective Helping Other. The dreamer remains the distant observer of an unbearable scene that overwhelms him, leading to a leading to a panicked awakening. This imagery reflects the patient’s early trauma: a state of radical helplessness, unmet appeals for rescue, and dissociation from intolerable affect.

In contrast, Dream 2 unfolds in an everyday interpersonal setting. No life-threatening scenario is present; instead, the dreamer encounters frustrating but tolerable situations involving negotiation, interruption, and social pressure. Affects are present, but they are no longer overwhelming, and the dream ends not in panic but in thoughtful reflection. This shift indicates that traumatic affect is no longer stored in a raw, unbound form but rather has become linked to symbolic and relational meaning.

##### 2.2.3.2 Transformation of dream organization and affective distribution

From a ZDPCS perspective, Dream 1 is dominated by the *security principle*, with the dream organizer attempting only to minimize affect and prevent disintegration. The traumatic dream complex is activated, but it cannot be processed; involvement is postponed because the emotional load remains intolerable.

Dream 2, by contrast, is not a nightmare and is structured through *affect induction.* Affective tension is distributed across multiple figures, reducing the emotional burden on the dreamer. This mechanism reflects improved integrative functioning: affect can now circulate within the interactive field rather than becoming concentrated in a single persecutory or fragmented object. The ability to tolerate distributed affective resonance signals a more resilient regulatory system.

##### 2.2.3.3 From passive observation to active participation and self-agency

In Dream 1, the dreamer witnesses the suffering of the injured man without being able to intervene. His pleas for help symbolize the patient’s unmet needs and the absence of a reliable internalized helper. The dreamer’s position—distant, fearful, and unable to act—captures his minimal self-agency at the beginning of treatment.

By contrast, in Dream 2, the dreamer actively participates in the unfolding events, asserts himself, and expresses annoyance and skepticism. He confronts others verbally, questions their intentions, and reacts with emotional clarity. These behaviors reflect significant gains in *self-agency*, boundary formation, and the ability to mentalize internal states and interpersonal meanings.

##### 2.2.3.4 Symbolization and reflexivity across time

The early dream shows little capacity for symbolization; affects appear in concrete, bodily form (such as exposed intestines), signaling that the traumatic experience remains unrepresented and psychically unintegrated. In contrast, Dream 2 employs symbolic elements – furniture, colleagues, and a funeral toast – to organize complex relational themes and emotional positions. Moreover, the dreamer’s waking thought, “What a strange dream,” demonstrates reflective distance and curiosity, qualities that were absent in the initial dream.

##### 2.2.3.5 Clinical implications: enduring effects of psychoanalytic treatment

Taken together, the two dreams illustrate long-term structural change. The early dream embodies the patient’s original state: dissociation, helplessness, absence of a regulating other, overwhelming trauma, and minimal agency. The second dream illustrates a reorganized internal world: affect is distributed, relational engagement is possible, self-agency is active, and internalized regulatory capacities are available even during new crises.

From both psychoanalytic and ZDPCS perspectives, the comparison of the two dreams provides evidence that psychoanalytic therapy can reorganize deep layers of traumatic memory and inner object-world representations – supporting enduring resilience even decades after treatment concludes.

## 3 Enduring change in psychoanalysis: dreaming and memory

The underlying argument of the clinical and dream material presented above is that Mr. P. displayed enduring change in his psychological functioning, which was supported by changes in memory processes. The questions then become: how do memory and dreaming play a role in the enduring changes in psychological functioning?

### 3.1 Dreams as potential indicators for memory retranscription

Memory has been central to psychoanalytic theory from the outset. As early as 1895, Freud claimed that any serious psychological theory must explain “memory” (Freud, 1895, p. 299). Soon after, he proposed that memory traces undergo “retranscription” (Umschrift) through successive “stratification” and “rearrangement” in light of new circumstances, so that “memory is present not once but several times over, … laid down in various kinds of indications (Zeichen)” [SIC] (Freud, 1896, p. 207). His later use of Umschrift in dream theory—“a mutilated and altered transcript (Umschrift) of certain rational psychical structures … ‘latent dream-thoughts’” [SIC] (Freud, 1905, p. 160)—highlighted how this retranscription entails active transformation, selection, and loss of material, as also implied in his account of “day’s residues” fueling dream work (Freud, 1905, pp. 160–161).

During transference, “embodied memories” of trauma are enacted (Leuzinger-Bohleber, 2015) and function as a Tagesrest that triggers dream work; analytic working-through places these memories in a labile state, enabling their transformation and yielding systematic changes in the content of manifest dreams. Mr. P.’s crisis intervention illustrates this: reactivated early separation anxieties, worked through via aggression, terror, and Oedipal interpretations, culminated in his “wild dream” featuring an overwhelming colleague/analyst once he again experienced the analyst as a Helping Other. The shifts between his first and second manifest dreams, 14 years apart, can thus be understood as clinical evidence of the ongoing retranscription processes of early, traumatic, embodied memories.

### 3.2 Memory reconsolidation and psychoanalysis

Lane et al. (2015, pp. 3ff.) argued that numerous neurobiological trauma studies corroborate the psychoanalytic concept of a dynamic unconscious: overwhelming traumatic experiences are multiply encoded, with some memory traces remaining unconscious yet powerfully shaping thought, affect, and behavior. Trauma occurs when emotional arousal exceeds available cognitive resources for processing (Lane et al., 2015, p. 5), and the authors proposed—converging with psychoanalytic views (e.g., Bohleber, 2012)—that previously unbearable affect must be re-experienced in psychotherapy to effect lasting change. Drawing on memory reconsolidation and the multiple trace theory (MTT; Moscovitch and Nadel, 1999; Nader, 2015), they emphasized that reactivation renders consolidated memories labile and modifiable, a process highly relevant clinically (Kim et al., 2021), given that new insights in analysis emerge on and blend with older traces. Lane et al. (2015, pp. 14ff.) thus presented an integrative memory model in which psychoanalytic treatments are especially suited to enduring character pathologies, as they directly accessed early, unconsciously acquired, embodied object relations encoded in procedural memory—an idea anticipated by Moser’s psychoanalytic research group in the 1990s, albeit without explicitly addressing (re)consolidation.

### 3.3 Dreams as memory processes

Freud’s hope “to furnish a psychology that shall be a natural science” (Freud, 1895, p. 295) can now be revisited partially through cognitive science research on dreaming and memory, a field too extensive to review here. For current overviews, see Bloxham and Horton (2024) on sleep/dreaming and memory consolidation, and Picard-Deland et al. (2023a) on memory reactivation as the neural basis of dreaming.

Bloxham and Horton (2024) grouped the indirect evidence linking dreaming and consolidation into three categories. First category suggests there are correlations between brain activity and dream content: 1) REM sleep and relaxed wakefulness show similar neural patterns, consistent with the hypothesis of a continuity between dreaming and waking mind-wandering (Bloxham and Horton, 2024, p. 2), and; 2) memory reactivation occurs in both sleep and quiet rest (Picard-Deland et al., 2023a), supporting a sleep–wake continuum of spontaneous thought. Second is evidence for the Continuity Hypothesis: up to 70% of dreams contain “day residue” (Nielsen and Powell, 1992), with early-night dreams emphasizing recent material, while later REM dreams draw on memories from 5–7 days earlier (the so-called “dream lag”: Blagrove et al., 2011) and even much older experiences (Vallat et al., 2017), suggesting the integration of new information into existing networks (Horton and Malinowski, 2015).

Third, there is more tentative evidence concerning the effects of dreams on waking behavior: the Enhancement Hypothesis posits that dream-based memory reactivation, especially of emotionally salient material, can support subsequent learning and recall (Bloxham and Horton, 2024). Conceptually, it remains unclear whether dreaming mediates consolidation or whether consolidation processes generate dream content; some reactivations may be deliberately incorporated into dreams while others covertly “prime” memories (Picard-Deland et al., 2023a), and waking cognition may tag certain memories for later sleep-based processing.

Although memory is involved, dreams rarely replay events accurately (Malinowski and Horton, 2014), except for some replicative trauma nightmares (Ross et al., 1989). More often, they recombine fragments of spatial, social, emotional, and sensory memories (Picard-Deland et al., 2023b) and can simulate future scenarios (Wamsley, 2022), with reactivation shaped by recency, novelty, and emotional intensity (Picard-Deland et al., 2023a). This supports a role for dreaming in integration, meaning-making, and emotional regulation.

Mr. P.’s clinical material is consistent with these findings: his worries about separation, grief, and illness, which led him to seek crisis intervention, are present in both his dreams and his waking life. This may indicate a sleep–wake continuum of spontaneous thought. The “day residue” of the analyst’s interruption during the session is presented in the dream. Finally, his free associations around films exemplify waking mind-wandering, and his dream blends recent and remote, social and emotional memories with low fidelity. Whether these operations specifically rely on memory reconsolidation, however, remains an open question.

## 4 Limitations

The limitations of conceptual, interdisciplinary work based on a single clinical case study are well-known and linked to interesting epistemological and methodological controversies. We were unable to discuss them in this study, but have addressed them in detail in other works on pluralism in contemporary psychoanalytic research (see, among others, Leuzinger-Bohleber et al., 2006; Leuzinger-Bohleber, in press). Depending on the epistemological position, for example, the generalizability of the considerations and hypotheses in conceptual research is questioned. Therefore, we would like to clarify that the connection we propose between psychoanalytic theory and the neuroscience concept of memory reconsolidation is primarily theoretical. We also hope that our study inspires other research groups to systematically test our hypotheses, for example, in an empirical-statistical design.

Another limitation concerns the theoretical integration of the perspectives presented here. With regard to this claim, too, we must point to our more extensive research on this matter.

## 5 Discussion and summary

In this article, we used a multiperspective single-case design to show how integrating clinical observation, dream analysis, and memory theory clarifies therapeutic change. Our findings and our attempt at theoretical integration illuminate how shifts in dreams and the inner world can be viewed as expressions of memory retranscription (Freud, 1896).

1. Activation of trauma in the transference.
  The case illustrates how Freud’s notion of memory retranscription converges with contemporary reconsolidation models: traumatic memories, reactivated in the transference, enter a new relational context in which they can be re-coded and reconsolidated. In Mr. P.’s analysis, early separation trauma was repeatedly revived and worked through with a reliable Helping Other, so that panic and death anxiety could be re-linked to new experiences; by the time of the crisis intervention, separation appeared in dreams as fear rather than catastrophic panic, suggesting this updating process. This supports the view that reactivating early trauma in transference is essential for deep change and highlights the time-intensive nature of this study (Bohleber and Leuzinger-Bohleber, 2016; Leuzinger-Bohleber, 2018), in line with Lane et al.’s emphasis on integrating safety, care, and compassion into traumatic memories via a “corrective emotional experience” (Lane et al., 2015, p. 5; Lane, 2018).^6^
  multiple trace similarly posits that each retrieval renders memories labile and open to modification (“an additional opportunity to amend…”: Lane et al., 2015, p. 12), so that old traces and affects are continually re-given meaning—akin to Freud’s Nachträglichkeit. In Mr. P., this process reshaped not only specific memories but also schemas and affective responses, illustrating how psychoanalysis can foster lasting transformation within a secure relational frame.
2. Session as dream/waking-dream equivalent.
  Research on dreaming and mind-wandering (Bloxham and Horton, 2024; Picard-Deland et al., 2023a) supports the idea that the analytic setting—with its regression, motor relative inhibition, and free association—induces a waking state similar to dreaming. As Bleger notes, the boundary between session and dream space is increasingly blurred (Bleger, 2021), and, as Rolland argues, the way a dream is spoken in session reveals its transference inscription, with analytic work extending dream work (Rolland, 2021, p. 55). Freud already conceived the analytic situation on the model of the dream (Freud, 1900, 1912): unconscious impulses “reproduce themselves” as if real, and the analyst’s task is to shift them from enactment to reflection. Contemporary authors conceptualize the session itself as a waking dream or oneiric field: dreams as thinking and self-regulation (Leuzinger-Bohleber, 1987, 1989, 2025; Gennaro et al., 2020; Roesler and Widmer, 2023; Angeloch, 2023); Ferro’s model of the session as an “oneiric holographic field” and “virtual reality” in which communications are treated as “I had a dream in which…” and transformed through “dreaming” (Ferro, 2002, 2019); Ogden’s idea of “dreaming oneself more fully into existence” within the analytic pair (Ogden, 2004, 2017). Empirical studies of dream series (Gennaro et al., 2020; Roesler and Widmer, 2023; Fischmann and Leuzinger-Bohleber, 2025) support the view that changes in dreaming parallel analytic transformation. Overall, many approaches now invite us to listen to the session as if it were a dream.
3. Dreams are more than memory performance.
  Picard-Deland et al. (2023a) argue that dreams arise from consolidation processes but go beyond them, contributing to meaning-making, emotional regulation, and future-oriented simulation. The ZDPCS-based analysis of Mr. P.’s second dream aligns with this perspective: it is no longer a nightmare, and shows improved affect regulation, attunement to others, and symbolic rather than raw affect expression. The dream thus exemplifies dreaming as a process of emotional regulation and meaning construction rather than mere memory replay.
4. Memory retranscription and reconsolidation.
  Memory retranscription and reconsolidation stem from different traditions and cannot simply be equated; for now, they function best as productive analogies. Reconsolidation offers a useful heuristic for psychoanalysis, much as Freud’s “Mystic writing-pad” did for theorizing the psychic apparatus (Freud, 1925).

In conclusion, analysands and poets venture into psychic regions often inaccessible to formal research. In Cymbeline, Posthumus’s dream of his unknown family is experienced as real, inscribing itself in memory “as though it were fact” and blurring the line between dream and reality — “‘Tis still a dream, or else such stuff as madmen tongue and brain not” (Shakespeare, 1988, pp. 239–240; Landry, 1982; Nolan, 2016). Dreams resist straightforward rational grasp and speak in their own idiom. The enigmatic interplay of dreaming and memory has long attracted clinicians and scientists alike; future work may clarify where poetic insight and empirical investigation converge.

## Footnotes

1 Since we lack the space to define terms such as basic trust, self-agency, trauma, and more in detail here, we refer you to: Ambresin et al. (submitted) (see also footnote 4).

2 For detailed clinical material on this patient, please see Ambresin et al. (submitted).

3 For changes to the manifest dream contents, and latent dream contents in the treatment of chronic depression, see Leuzinger-Bohleber (1987, 1989), Fischmann et al. (2012), and Fischmann and Leuzinger-Bohleber (2025).

4 Today, the term trauma is often used in an inflationary manner and thus loses its specific explanatory content. For this reason, following Cooper (1986), the authors [MLB] used the definition of trauma in the narrower sense: “A psychic trauma is an event which abruptly overwhelms the abilities of the ego to ensure a minimal sense of security and integrative completeness, and which leads to an unbearable anxiety or sense of helplessness, or else leads to the threat of this; it leads, furthermore, to an enduring change of the psychic organization” (p. 44). This definition includes both psycho-economic and object-relations theoretical perspectives, as Bohleber (2012) also points out: “Psychoanalytic trauma theories have evolved on the basis of two models, one psycho-economic, the other hermeneutic and based on object relations theory. To grasp the phenomenology and long-term consequences of trauma, we need both models. The psycho-economical model focuses on excessive arousal and on anxiety that cannot be contained by the psyche and that breaks through the shield against stimuli. The model based on object relations and the breakdown of internal communication which produces an experience of total abandonment, precluding the integration of trauma by narrative means” (xxi) [SIC].

5 One central topic of the last analytic session was that Mr. P. had great success in his job (as an engineer).Even his difficult boss praised him. Since he could only report casually on his success during the session, it was possible to address a central aspect of his underlying depression: out of fear of the destructive envy of his objects and the murderous aggression this triggers in him, he must renounce success, or destroy success itself, or at least deny it.

6 The term “corrective emotional experience” was first brought into discussion in psychoanalysis by Alexander and French (1946). He challenged the techniques of abstinence, neutrality, and anonymity by postulating that “the analyst does not interpret the patient’s transference, but instead purposefully adopts attitudes aimed to counter the presumed pathogenic attitudes of the patient’s original objects” [SIC]. In response, Eissler (1950) argued that interpretation is the model technique of psychoanalysis and that modifications of that technique that might include more gratifications of that technique are “’parameters’ which should have only limited, temporary use and whose impact should be analyzed” (Auchincloss et al., 2012, p. 2). To this day, in psychoanalysis, the term “corrective emotional experience” is highly controversial. For this reason, we prefer to speak about *alternative object-relational experiences* of the patient during analytic sessions, which, of course, also become part of their embodied memories and can be used in a productive way in the above-mentioned regained self-agency and self-reflection processes (see Section 3.1).
